# Increase in Registered Acute Kidney Injuries in German Hospitals

**DOI:** 10.7759/cureus.36868

**Published:** 2023-03-29

**Authors:** Ludwig Matrisch, Hendrik Karsten, Justus Schücke, Yannick Rau

**Affiliations:** 1 Nephrology, University of Lübeck, Lübeck, DEU; 2 Infectious Disease, University of Hamburg, Hamburg, DEU; 3 Surgery, University of Lübeck, Lübeck, DEU

**Keywords:** germany, gender disparities, gender, epidemiology, incidence, acute kidney injury

## Abstract

Background: Currently, the development of the incidence of acute kidney injury (AKI) and the influence of age and gender on the condition in Germany is unclear.

Materials and Methods: Data were extracted from the national database of Federal Health Reporting. It was then normalized for demographic changes. Poisson regression was performed on 933,684 cases to quantify the correlation between age, years, and AKI incidence. Analysis of variance was performed on the same collection to evaluate gender disparities in different age groups.

Results: In absolute numbers, registered AKI increased almost sevenfold from 11,964 to 77,719 between 2000 and 2019. After adjusting for demographic changes, the most AKI - 6300.5 per million person-years - occur in the elderly (>79 years old). Males have a higher risk for the development of an AKI. The male and female AKI incidence ratio varies significantly between different age groups, and it is the lowest in people <20 and >79 years old.

Conclusions: The registered incidence of AKI has risen substantially in the first 20 years of the millennium. The increase can partly be attributed to an increased diagnostic sensitivity provided by changes in the classification of AKI. It could also be shown that men suffer from AKI more often than women, particularly in the younger age groups.

## Introduction

Acute Kidney Injury (AKI) is possibly the most important clinical condition in nephrology due to its devastating effect on kidney function and patient health [1]. AKI leads to increased morbidity and mortality in acute situations and often requires renal replacement therapy (RRT) to deal with the consequences [2]. It also accelerates the progression of chronic kidney disease (CKD) [3]. Patients with AKI are at increased risk for developing CKD and end-stage kidney disease (ESKD) [4]. They are also more prone to cardiovascular events, increasing long-term mortality [5].

Therefore, AKI places a massive burden on the healthcare system and is associated with increased healthcare costs in acute and long-term situations. AKI, in the acute situation alone, accounts for just over 1% of the National Health Service budget in Great Britain [6]. In the USA, the annual costs are estimated at up to $24.0 billion [7]. Unfortunately, no data is readily available for the long-term impact of AKI on the healthcare system in Germany specifically. However, it is well known that the progression of CKD leads to increased healthcare costs. Gandjour et al. estimate those costs at 8,030€ per year for patients with stage 3 CKD, 9,760€ in stage 4 CKD, and even 44,374€ in stage 5 CKD [8]. Compared to an age- and gender-matched control group, this amounts to a 179%, 239%, and 1443% increase, respectively [8].

Due to its importance, the incidence of AKI has already been the subject of various studies. However, these studies mainly focus on AKI in patients already hospitalized before the onset of the AKI. This is an essential field of study but needs to be generalized to the population. Reliable incidence data, however, is crucial for health care policies and has implications for allocating medical resources such as nephrologists and machines for RRT. Therefore, we concentrated our analysis on the incidence in the general population instead.

In this paper, we use a registry-based approach to quantify the incidence of AKI in Germany from 2000 to 2019. We also research gender disparities in different age groups and elaborate on their potential causes. Furthermore, we explore the possible influence of changing diagnostic criteria for AKI on the registered occurrences and compare the development in Germany to that in other countries.

## Materials and methods

Data collection

AKI incidence data were retrieved from the Gesundheitsberichterstattung des Bundes (Federal Health Reporting) database of the federal statistical office, Department of the Interior. In the German healthcare system, every hospital treating patients insured by statutory health insurance must report specific data - including diagnoses - to state institutions. These data are then bundled in the Federal Health Reporting database. The data for the International Statistical Classification of Diseases and Related Health Problems (ICD) 10 - German Modified code N.17 - which codes for AKI - were retrieved and prepared for further analysis.

Demographic data were retrieved from the GENESIS database of the federal statistical office of Germany. The GENESIS database is the hub of the official statistics kept by the state offices of statistics as well as the federal statistics office.

Statistical analysis

Statistical analysis was performed using Microsoft Excel (Version 2207, Microsoft, Redmond, USA) and Jamovi (Version 2.2.5, The Jamovi Project, Sydney, Australia). Incidence data were transformed into incidence per million person-years (pmpy) using population data for accounting for demographic changes. Poisson regression analyses were performed to quantify the relationship between the years and AKI incidence. Additionally, analysis of variance (ANOVA) was performed to compare the gender disparities between different age groups using the male-to-female incidence ratio as the dependent variable and the age groups as fixed factors. Significance level α was set at 0.05, as is a custom in medical research.

## Results

Absolute numbers

**Figure 1:**
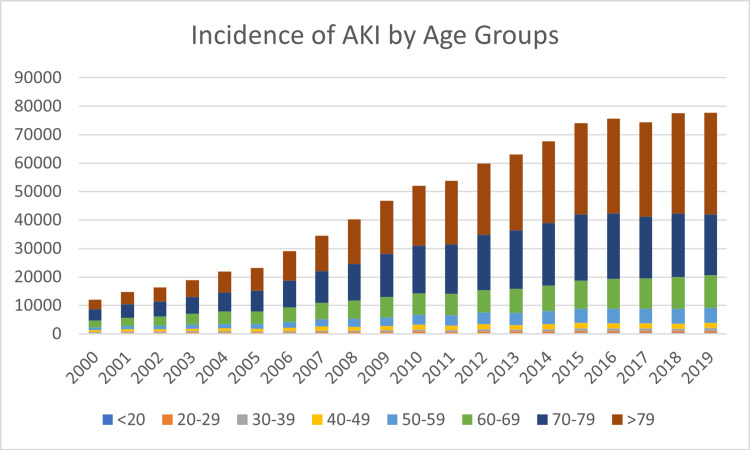
Absolute Incidence of AKI by Age Groups AKI: Acute kidney injury

Registered AKI has increased significantly from 11964 cases in 2000 to 77,719 in 2019. The total number of registered AKI in the investigated period was 933,684. Figure 1 exhibits the development of the incidences subdivided into age groups. While back in 2000, the group of patients between 70 and 79 made up the biggest group of AKI patients; this changed in 2004. From 2004 up until 2019, the elderly (>79) had the largest share of AKI patients.

Relative numbers

**Figure 2:**
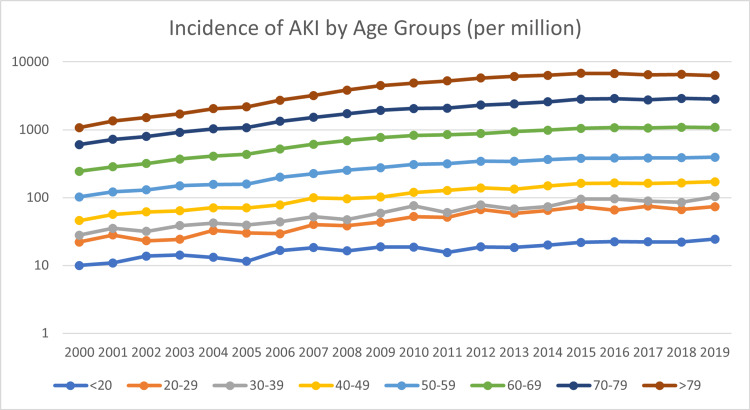
Incidence of AKI per million person-years; logarithmic scale AKI: Acute kidney injury

The German population's average age increased between 2000 and 2019, which impedes comparing the years. The comparison with other countries with different demographic properties is also hindered. To account for that, we report the relative incidences per million person-years. The results are presented in table 1 and figure 2 using a logarithmic scale. Figure 2 illustrates the higher relative than absolute share of patients over the age of 79 in AKI cases. This can be attributed to the smaller number of people in this age group compared to other groups.

**Table 1:** AKI incidence per million person-years AKI: Acute kidney injury

year | age	<20	20-29	30-39	40-49	50-59	60-69	70-79	>79	Overall
2000	9.95	22.14	27.94	45.77	102.5	244.15	603.53	1076.24	145.44
2001	10.83	28.1	35.22	56.27	121.29	284.57	720.953	1349.85	178.76
2002	13.75	23.11	31.64	61.34	129.43	317.55	796.71	1510.1	198.8
2003	14.2	24.21	38.5	63.9	149.33	369.68	915.3	1704	229.2
2004	13.16	32.69	41.96	70.99	155.90	408.50	1026.58	2041.9	265.02
2005	11.46	29.98	39.25	70.39	157.83	434.87	1074.42	2166.64	281.54
2006	16.54	29.38	43.98	77.85	198.82	522.27	1326.88	2723.89	353.08
2007	18.27	40.07	51.94	99.25	225.15	609.72	1520.31	3197.75	420.31
2008	16.39	38.24	47.11	95.93	252.33	688.66	1716.79	3845.5	491.35
2009	18.71	43.48	58.85	101.74	275.42	767.84	1937.62	4459.55	571.74
2010	18.63	52.48	75.64	119.12	307.82	825.02	2057.33	4872.68	636.01
2011	15.54	51.05	59.94	126	314.6	846.19	2075.57	5245.98	670.48
2012	18.75	66.75	77.88	139.13	343.09	874.72	2306.81	5765.75	743.04
2013	18.47	58.40	67.87	132.79	342.24	940.0	2402.34	6110.35	781.34
2014	19.93	64.49	73.32	148.66	363.34	982.55	2576.05	6322.21	834.41
2015	21.83	74.08	95.06	162.15	379.89	1045.53	2824.58	6769.22	901.56
2016	22.34	65.19	95.53	163.60	380.47	1070.74	2871.13	6745.57	916.94
2017	22.29	74.64	88.58	161.67	382.96	1058.71	2751.89	6441.86	897.59
2018	22.1	66.63	84.82	165.06	386.96	1086.83	2898.54	6544.31	934.75
2019	24.4	73.33	102.55	170.59	394.27	1081.97	2824.71	6300.50	934.5

The general trend of an increase in AKI cases can also be observed here. Poisson regression was performed to quantify this rise. Model fitness proved excellent as measured by the loglikelihood ratio test (Χ2=3082, df=19, p<0.001). The results are presented in table 2.

**Table 2:** Poisson regression analysis of relative AKI incidence in dependence on the calendar year Abbreviations: SE=standard error; exp(B)=exponentiated beta coefficient; AKI: Acute kidney injury

Effect	Estimate	SE	exp(B)	z	p
(Intercept)	6.182	0.0112	483.79	550.02	<0.001
2001 - 2000	0.206	0.1117	1.23	1.85	0.065
2002 - 2000	0.313	0.1091	1.37	2.86	0.004
2003 - 2000	0.455	0.1060	1.58	4.29	<0.001
2004 - 2000	0.600	0.1032	1.82	5.81	<0.001
2005 - 2000	0.661	0.1021	1.94	6.47	<0.001
2006 - 2000	0.887	0.0985	2.43	9.00	<0.001
2007 - 2000	1.061	0.0962	2.89	11.03	<0.001
2008 - 2000	1.217	0.0944	3.38	12.90	<0.001
2009 - 2000	1.369	0.0929	3.93	14.74	<0.001
2010 - 2000	1.475	0.0919	4.37	16.05	<0.001
2011 - 2000	1.528	0.0915	4.61	16.71	<0.001
2012 - 2000	1.631	0.0907	5.11	17.99	<0.001
2013 - 2000	1.681	0.0903	5.37	18.62	<0.001
2014 - 2000	1.747	0.0899	5.74	19.44	<0.001
2015 - 2000	1.824	0.0894	6.20	20.42	<0.001
2016 - 2000	1.841	0.0893	6.30	20.63	<0.001
2017 - 2000	1.820	0.0894	6.17	20.36	<0.001
2018 - 2000	1.860	0.0891	6.43	20.87	<0.001
2019 - 2000	1.860	0.0891	6.43	20.87	<0.001

Gender disparities

**Figure 3:**
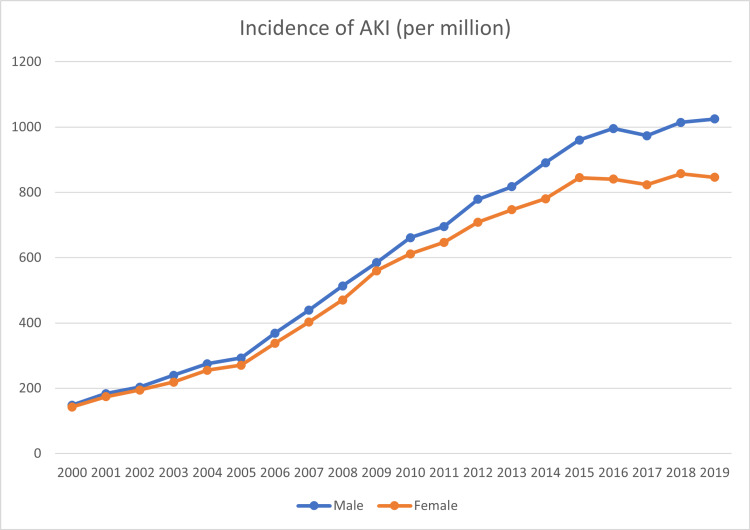
AKI Incidence per million person-years divided by gender AKI: Acute kidney injury

Stratification of patients into a male and a female group revealed significant differences in the incidence of AKI between the two genders. AKI is more common in males, as shown in figure 3.

**Figure 4:**
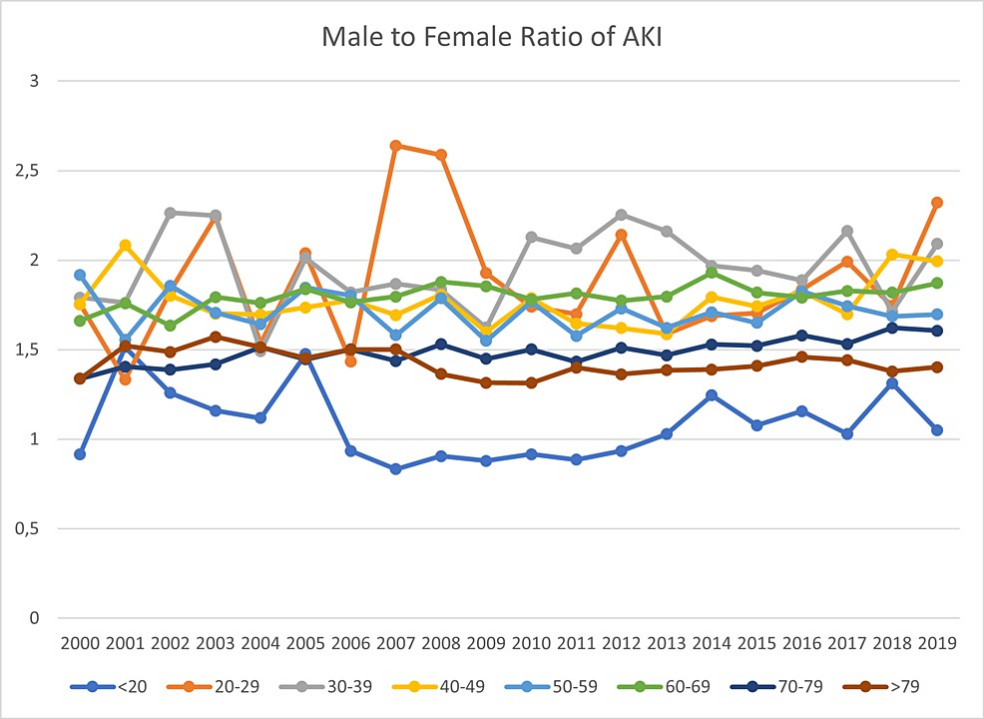
Development of the Male to Female Ratio of AKI Incidence AKI: Acute kidney injury

These gender disparities vary between different age groups. While the ratio of AKI between male and female adolescents (<20 years) oscillated around 1 between 2000 and 2019, it was higher in young and middle-aged adults. In 2007, males between 20 and 29 had a 163.96% elevated risk of suffering from AKI compared to females of the same age group. However, the ratio was volatile, and in the year just prior the risk was only elevated by 43.39%. These numbers vary a little during the period analyzed in this paper. The exact development of these numbers is presented in figure 4.

The results of an ANOVA to check for differences in the gender disparities between the age groups are presented in table 3. The male-to-female ratio of AKI incidence was the dependent variable, and the age group was a fixed factor. Before the ANOVA, Levene's test of equal variances was performed (F=11.7, df1=1,df2=152, p<0.001). The ANOVA shows excellent model fitness (sum of squares=11.78, df=7, mean square=1.6824, F=52.4, p<0.001). This analysis further supports the notion of significant differences in the male-to-female ratio between the analyzed age groups, which can also be observed in figure 4. These differences are only sometimes significant when comparing adjacent age groups, most likely due to the similarity in age between these groups.

**Table 3:** Gender Disparities; Analysis of Variance between different age groups Abbreviations: SE=standard error; df=degrees of freedom

age group	compared to: age group	mean difference	SE	df	t	p
<20	20-29	-0.8069	0.0567	152	-14.235	<0.001
	30-39	-0.8727	0.0567	152	-15.396	<0.001
	40-49	-0.6870	0.0567	152	-12.120	<0.001
	50-59	-0.6312	0.0567	152	-11.136	<0.001
	60-69	-0.7169	0.0567	152	-12.647	<0.001
	70-79	-0.4050	0.0567	152	-7.145	<0.001
	>79	-0.3439	0.0567	152	-6.067	<0.001
20-29	30-39	-0.0658	0.0567	152	-1.161	0.247
	40-49	0.1199	0.0567	152	2.115	0.036
	50-59	0.1757	0.0567	152	3.099	0.002
	60-69	0.0900	0.0567	152	1.589	0.114
	70-79	0.4019	0.0567	152	7.091	<0.001
	>79	0.4630	0.0567	152	8.168	<0.001
30-39	40-49	0.1857	0.0567	152	3.276	0.001
	50-59	0.2415	0.0567	152	4.260	<0.001
	60-69	0.1559	0.0567	152	2.750	0.007
	70-79	0.4677	0.0567	152	8.252	<0.001
	>79	0.5288	0.0567	152	9.329	<0.001
40-49	50-59	0.0558	0.0567	152	0.984	0.327
	60-69	-0.0298	0.0567	152	-0.527	0.599
	70-79	0.2820	0.0567	152	4.976	<0.001
	>79	0.3431	0.0567	152	6.053	<0.001
50-59	60-69	-0.0856	0.0567	152	-1.511	0.133
	70-79	0.2263	0.0567	152	3.992	<0.001
	>79	0.2873	0.0567	152	5.069	<0.001
60-69	70-79	0.3119	0.0567	152	5.502	<0.001
	>79	0.3729	0.0567	152	6.580	<0.001
70-79	>79	0.0611	0.0567	152	1.077	0.283

## Discussion

Classification

It is important to note that only patients who fell under the AKI criteria valid at admission could be included in this study. With this in mind, one can assume that portions of the increase seen in the period result from more patients with impaired kidney function and a further adaptation of more and more sensitive diagnostic criteria for the classification of AKI [9].

At the beginning of the investigated period in 2000, there were no globally accepted AKI classification criteria. More than 35 different definitions have been used in the literature. This changed after the second international consensus conference of the Acute Disease Quality Initiative in Vicenza, Italy, and its results were published in 2004 [10]. From then on, these criteria - known as the RIFLE criteria - became a widely accepted gold standard worldwide. They were superseded by criteria published in 2007 by the Acute Kidney Injury Network [11]. These criteria increased the diagnostic sensitivity of the detection of an AKI due to lower threshold levels for clinical and laboratory parameters. The guidelines published by the Kidney Disease: Improving Global Outcomes (KDIGO) network in 2012 are commonly used in clinical practice [12]. The sensitivity of detecting an AKI was increased even further due to longer timeframes for the diagnosis. The criteria are presented in table 4.

**Table 4:** Criteria for the classification of acute kidney injury Abbreviations: RIFLE= Risk of renal dysfunction, Injury to the kidney, Failure of kidney function, Loss of kidney function and End-stage kidney disease; AKIN= Acute Kidney Injury Network; KDIGO= Kidney disease: improving global outcomes; MDRD= modification of diet in renal disease; SCreat= Serum creatinine; GFR= glomerular filtration rate; RRT= renal replacement therapy Sources: RIFLE criteria [10]; AKIN criteria [11]; KDIGO criteria [12]

Criteria	RIFLE	AKIN	KDIGO
Year of release	2004	2007	2012
Baseline	Not specified. In case of unknown baseline GFR, the MDRD equation is recommended.	48 h-window; not specified in case of unavailability of prior values	Not specifically defined. If unavailable, the lowest SCrea measured during hospitalization or the MDRD equation should be used.
Time Interval	1-7 days both for diagnosis and staging	48 hrs for diagnosis and 7 days for staging	Diagnosis: 48 hrs or 7 days, respectively
Stage I	Increased SCreat x1.5 or GFR decrease >25% OR urine output <0.5 ml/kg/h x6 hrs	Increased SCreat x1.5 or ≥ 0.3 mg/dl OR urine output <0.5 ml/kg/h x6 hrs	Increased SCreat x1.5 (7 days) or ≥ 0.3 mg/dl (48 hrs) OR urine output <0.5 ml/kg/h x6 hrs
Stage II	Increased SCreat x2 or GFR decrease >50% OR urine output <0.5 ml/kg/h x12 hrs	Increased SCreat x2 OR urine output <0.5 ml/kg/h x12 hrs	Increased SCreat x2 OR urine output <0.5 ml/kg/h x12 hrs
Stage III	Increased SCreat x3 or GFR decrease >75% OR urine output <0.3 ml/kg/h x24 hrs or anuria x12 hrs	Increased SCreat x3 or over 4 mg/dl with an acute increase ≥ 0.5 mg/dl OR urine output <0.3 ml/kg/h x24 hrs or anuria x12 hrs	Increased SCreat x3 or over 4 mg/dl or in patients < 18 years a decrease in GFR <35 ml/min/1.73 m² OR initiation of RRT OR urine output <0.3 ml/kg/h x24 hrs or anuria x12 hrs
Notable differences	-	1: Time window for diagnosis reduced to 48 hrs 2: addition of 0.3 mg/dl absolute change in SCreat as diagnostic criterion 3: GFR criteria removed	1: new GFR criterion for children 2: addition of RRT as criterion 3: timeframes for diagnosis 4: 0.5 mg/dl no longer required in stage III

All three classifications use Serum Creatinine (SCreat) and Urinary Output (UO) as criteria for diagnosing an AKI. While in the RIFLE classification, patients required a SCreat rise of 50% or a drop of 25% of their glomerular filtration rate (GFR), the latter criterion was dropped by the AKIN consortium in favor of a rise of SCreat of 0.3 mg/dl. Especially in patients with an already impaired GFR - a preexisting condition highly prevalent in AKI patients -this increased the diagnostic sensitivity [13].

Whether a condition classifies as AKI under either framework is dependent on the baseline SCreat value used to determine the SCreat increase. Ideally, one would choose SCreat values measured right before the AKI onset. Unfortunately, there are many cases where these ideal SCreat values are unavailable. The RIFLE classification and the KDIGO classification recommend using the modification of diet in renal disease (MDRD) equation to estimate the baseline SCreat in these cases [14]. The AKIN classification does not specify their recommendation on estimating the baseline SCreat; however, one can assume that the MDRD equation is often used. Using a baseline SCreat estimated by the MDRD equation, however, has been shown to significantly overestimate the incidence of AKI in multiple studies on large patient populations [15]. This overestimation happened especially within patients in higher stages of CKD since the MDRD equation estimates the GFR to be around 75 ml/min/1.73 m². Patients with an initially lower GFR and, therefore, higher SCreat would clinically present like an AKI without any change to their renal function if the baseline renal function was estimated too high.

We purposefully only concentrate on the increased sensitivity of AKI stage I, as these patients already qualify for AKI reporting. This might raise a bit of confusion since the German term for AKI is "Akutes Nierenversagen", which literally translates to "acute renal failure". However, it is used synonymously with AKI, and a court has confirmed that it should be coded and reported that way [16].

Looking at the data, it seems plausible that the significant increase in registered AKI is connected to the increased diagnostic sensitivity of the classification criteria and, therefore, likely represents the gradual adoption of these classifications.

Usually, one would expect an instantaneous rise in reported AKI right after the publication of the classifications. However, it takes some time for the new guidelines to be implemented into the daily clinical routine. Unfortunately, we need help finding data on how quickly guidelines or novel classification systems are implemented in our thorough literature review. This calls for more research to find strategies to improve these implementations eventually. Although, flattening the curve after 2016 implies an almost complete saturation of diagnostic criteria.

Comparison to other countries

A similar rise in the incidence of AKI has already been described in other countries. Bien et al. reported an increase in primary and secondary AKI diagnoses in England between 1998 and 2020 [17]. Similar findings were made by Kolhe et al. [18]. At the same time, the fraction of AKI treated with RRT had decreased, leading the authors to conclude that the increase resulted from increased sensitivity of diagnostic criteria. In the USA, Pavkov et al. found an increase of registered AKI by 139% in patients with diabetes and 230% in patients without diabetes between 2000 and 2014 [19]. However, Kashani et al. assessed the incidence of AKI between 2006 and 2014 using an electronic surveillance tool applying the same diagnostic criteria for AKI throughout the years and found no significant increase after adjusting for age and sex [20]. These discrepancies could also be explained by the increased sensitivity of the diagnostic criteria described in this article.

Gender disparities

Our data indicate male gender is a risk factor for the development of AKI. This has already been described in various epidemiologic studies [21]. The age dependency of the gender disparities is much less described. Our data show almost no differences in the incidence of AKI between males and females under the age of 20, followed by a period of a risk elevated by 88,88% on average for men to develop AKI compared to women between 20 and 29 years. In the later age periods, the male-to-female ratio declines but constantly stays above one. This indicates either female-specific protective factors or male-specific risk factors, starting roughly with the onset of adulthood. The literature has already discussed the renoprotective effect of estrogen and could explain some of the differences [22]. These findings could also be shown in animal models [23]. Estrogen levels as the main protective factor would implicate a decrease in gender disparities at around 50 years of age due to the onset of menopause in women [24]. Contradictory to this assumption, our data show a persistently increased male-to-female ratio in older age groups, indicating other important factors at work. Also, a legacy effect of the renoprotective effects of estrogen cannot be ruled out.

Well-known risk factors for community-acquired AKI are sepsis, CKD, and diabetes [25]. Sepsis is more common in men [26]. Conversely, CKD is more prevalent among women [27]. Diabetes is more prevalent in men until age 80 [28]. Additionally, Hoste et al. found hypertension and liver failure to be risk factors for AKI in hospitalized patients [29]. Both factors are especially prevalent in males and could be other causes for the gender disparities in AKI incidence [30,31]. The direct and indirect influence gender has on the risk of AKI is highly complex and warrants further research to advance gender-specific personalized care and prevention. Given that lifestyle choices can influence some of the underlying diseases, one can conclude that the gender disparities described in this paper are partly due to natural causes but can and should also partly be influenced by improved health behavior.

Advantages and disadvantages of our method

This study is the first to provide reliable data for the incidence of AKI in all of Germany. Although the field of AKI has been the subject of epidemiological research before, this has yet to be accomplished. We propose a novel method of quantifying the incidence of severe diseases by using nationwide hospital-reported data. However, this causes some problems: First, the data only include patients treated in hospitals, since only those - unlike private practices or ambulatory nephrology institutes - report their data to Federal Health Reporting. That excludes patients with AKI treated in outpatient clinics and those who do not see a doctor.

Nonetheless, these cases are rare due to Germany's well-developed primary health care system and the often severe nature of the disease. Also, the proportion of patients with AKI not being treated in a hospital will likely stay the same within our observation period. Therefore, this would not influence the general trend demonstrated in this study.

The numbers presented here could also be inaccurate due to the nature of the federal health reporting system. Patients and their cases are reported only after their discharge. If a patient were to be admitted at the end of one year and discharged at the beginning of the following one, their AKI would count towards the second year. Since this occurs at every turn of the year, the effects will likely worsen. Also, underreporting of AKI must be considered.

## Conclusions

An apparent and drastic increase in registered instances of AKI between 2000 and 2019 in Germany is evident, and similar findings have been seen in other countries. However, to what extent this increase reflects worse renal health or rather only more awareness and more sensitive diagnostic criteria remains to be determined. The higher sensitivity of the current KDIGO AKI classification system should be considered when comparing current outcomes to those of earlier classification systems. Due to the improved classifications, more AKI is noticed, and patients can profit from adequate therapy. The clinical implications of shifting diagnostics criteria and the following effects on patient outcomes and public health measures, such as disease-specific healthcare spending, morbidity, and mortality, necessitate further research.
